# Recurrent Tachycardia, Abdominal, and Chest Pain as a Presentation of Stiff Person Syndrome

**DOI:** 10.1155/carm/4821987

**Published:** 2025-08-03

**Authors:** Neeki Torabi, Padi Reddy, Amir Torabi

**Affiliations:** ^1^University of the Incarnate Word, San Antonio, Texas, USA; ^2^Prime Healthcare, Department of Internal Medicine, Dallas Regional Medical Center, Mesquite, Texas, USA; ^3^Prime Healthcare, Department of Neurology, Dallas Regional Medical Center, Mesquite, Texas, USA

**Keywords:** anti GAD, spasms, stiffness, stiff person syndrome

## Abstract

**Introduction:** Stiff person syndrome (SPS) is a rare neurological disorder marked by muscle stiffness, spasms, specific electromyographic findings, and elevated levels of glutamate acid decarboxylase. Patients' symptoms and signs can be challenging for general practitioners and specialists.

**Case report:** We present a case of a 56-year-old man with a history of type 1 diabetes with episodes of severe chest, abdominal, and low back pain; severe tachycardia; and difficulty with walking who was seen by different physicians over a period of 10 months without any significant improvement. He had significant weight loss during this period due to abdominal pain. Multiple studies, including computerized tomography and magnetic resonance imaging of the abdomen and entire spine, upper and lower gastrointestinal (GI) endoscopies, and cardiac catheterization, were unremarkable. The patient presented at our facility with severe abdominal and chest pain, diffuse abdominal muscle rigidity, and periods of severe tachycardia. He also had elevated creatine kinase and lactate levels. Extensive workup for infectious, cardiac, and GI processes was negative. The patient was diagnosed with SPS based on history, clinical examination, and an exceedingly high titer of glutamic acid decarboxylase. He responded well to oral diazepam, baclofen, and gabapentin, and he received a 5-day course of intravenous immunoglobulin therapy.

**Conclusion:** In patients presenting with recurrent tachycardia, abdominal pain, and chest pain, SPS should be considered in the differential diagnosis. It is essential for non-neurologists to be familiar with this disorder.

## 1. Introduction

Stiff person syndrome (SPS) was described initially by Moersch and Woltman at the Mayo Clinic in 1956 [1]. The biological basis of muscle stiffness and spasm is related to autoimmune hyperexcitability caused by impaired gamma amino butyric acid (GABA)ergic inhibition, which explains the therapeutic response of GABAergic medications [2]. Glutamic acid decarboxylase (GAD) is the enzyme catalyzing synthesis of GABA; anti-GAD antibody (GAD Ab) will lower the level of GABA in the central nervous system (CNS).

There is a spectrum of positive GAD antibody neurological manifestations including SPS, cerebellar ataxia, autoimmune epilepsy, limbic encephalitis, and progressive encephalomyelitis with rigidity and myoclonus (PERM). The frequency seems to be one per million, but the actual number could be higher because of a lack of accurate diagnosis. Diagnosis of SPS is based on stiffness, muscle spasm, electromyography (EMG) findings (continuous motor unit firing at rest of both agonist and antagonist muscles), and high titer of GAD 65 Ab serology, while excluding other causes [2, 3]. Stiffness of axial and proximal muscles, especially abdominal and thoracolumbar paraspinals can cause a freezing-like appearance and uncontrolled falls. Painful muscle spasms can be misdiagnosed as anxiety attacks or phobias [4]. Stiffness can affect only one limb (stiff limb syndrome) or the face [5]. SPS has a highly elevated GAD Ab level > 2000 IU/mL in serum or CSF. Diabetes type 1 can cause elevated titers of GAD antibodies. A negative GAD antibody does not exclude the possibility of SPS. Antibodies against glycine receptors can be seen in patients with PERM. Amphiphysin antibodies are positive in 5% of patients with the paraneoplastic form of SPS [6]. Gastrointestinal (GI) symptoms can be seen in 32% of the patients, including dysphagia, constipation, nausea, and vomiting [7]. Severe pain and spasm can cause tachycardia which can confuse the physician. Another cause of tachycardia in SPS is dysautonomia [8]. Elevated creatine kinase (CK) can be seen with SPS, like myopathies or myositis [9]. Treatment includes GABAergic medications such as diazepam 5–10 mg twice a day, and if needed, 10 mg three times a day. Clonazepam is also effective at 0.5–1 mg three times a day. Baclofen (another GABAergic medication) starts at 10 mg three times a day and can be increased slowly to 20 mg three times a day. Gabapentin (although it is not a GABAergic medication, it may increase the expression of GABA A receptors) at 300 mg three times a day and can be increased slowly to 900 mg three times a day will help for pain and spasm. If there is no improvement after 2-3 months of the above medications, IVIG at 2 g/kg per month divided over 2-3 consecutive days shows a significant reduction of stiffness and flexibility in 75% of the patients [2, 10].

## 2. Case Presentation

**Figure 1:**
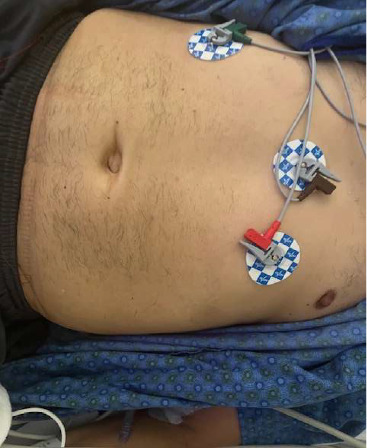
Abdominal muscles rigidity.

**Figure 2:**
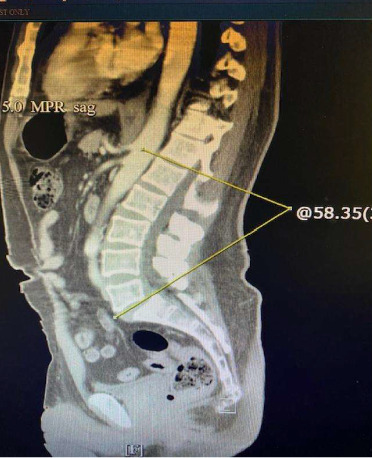
Hyperlordosis of lumbar spine.

The patient is a 56-year-old right-handed Hispanic male with a past medical history of type 1 diabetes, who presented with a 10-month history of refractory and recurrent severe chest and abdominal pain, low back pain, diarrhea, severe weight loss of 30 kg, and difficulty with walking during episodes. He was seen by multiple specialists and general practitioners. Symptoms started in October 2023 with abdominal pain and rigidity. The patient was seen at the emergency room by a general surgeon for the possibility of peritonitis, which was excluded. The patient's symptoms have worsened over time and he was seen by gastroenterologists. Multiple upper and lower GI endoscopies were negative for any evidence of polyp or cancer. He was diagnosed with gastroesophageal reflux disease, but treatment of reflux was not helpful for patient symptom alleviation. He also had severe back pain and was evaluated by a neurosurgeon. MRI of the entire spine showed nonspecific degenerative changes although paraspinal muscles T2 hyperintense signal was noted. The neurosurgeon suggested pain medication including narcotics and physical therapy. Because of severe episodes of tachycardia and chest pain, he also was evaluated by multiple cardiologists. CT angiography of the heart and cardiac catheterization were performed but were unrevealing. Since there was no obvious explanation of his symptoms, his physicians thought all his symptoms were functional, and the patient was diagnosed with a possible generalized anxiety disorder. Interestingly, when lorazepam was given for this possibility, his symptoms improved to some degree, making his physicians more confident the symptoms are all related to possible underlying anxiety or depression. After 10 months of recurrent symptoms, he presented to the emergency department with severe abdominal and chest pain and an inability to walk. On the physical examination, the patient had an apprehensive face, tachycardia up to 170 per minute, very rigid abdominal muscles (Figure 1), stiffness of lower extremities muscles, and significantly impaired walking (afraid of falling due to stiffness). There was no startle response. Deep tendon reflexes were brisk, and the Babinski reflex was negative. Sensory examination was intact. On his laboratory data, his CK was elevated at the level of 1100 U/L (normal: 53–446 U/L), and his lactate level was increased at 8 mmol/L (normal: less than 2 mmol/L). Because of the increased lactate level, a workup for infectious diseases was done and was negative. The general surgeon did not find any surgical lesion causing his abdominal pain. CT and MRI of the abdomen did not show any intra-abdominal pathology but showed T2 hyperintensities of paraspinal muscles and increased lumbar lordosis at 58° (normal: 25–45°) (Figure 2).

**Figure 3:**
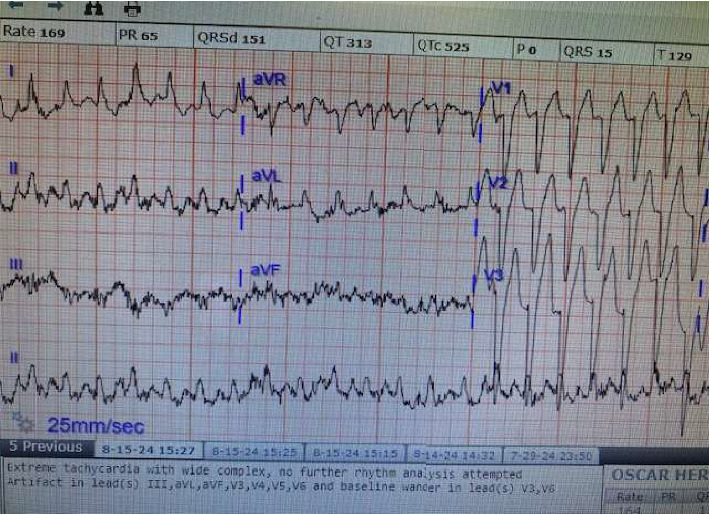
Tachycardia due to dysautonomia vs. pain.

The patient was evaluated by a cardiologist because of chest pain and severe tachycardia (Figure 3) up to 170/min. He was started on metoprolol 50 mg twice a day that was increased to 100 mg bid. CT angiography of the chest was negative. Checking for 24-h urine metanephrines, 5 hydroxyindoleacetic acid (5-HIAA) and vanillylmandelic acid (VMA) were normal. B12 level was normal and antinuclear antibody (ANA) were negative. Tumor markers, including carcinoembryonic antigen (CEA) and CA 19-9, were negative. Workups for possible malignancy, including CT scans of the chest, abdomen, and pelvis, were negative. Because of high suspicion of SPS, serum GAD-65 Ab level was requested, and it was diagnostic at a high titer of GAD > 25,000 IU/mL (normal < 5 IU/mL). EMG could not be done at the hospital and was scheduled to be done as an outpatient. Based on clinical exams, including typical muscle spasm, stiffness of thoracoabdominal and proximal muscles, and highly elevated GAD-65 antibody, as well as excluding other neurological and systemic disorders, he was diagnosed with SPS. Lumbar puncture was not done. He responded well to diazepam 5 mg 3 times a day, with significant reduction of spasm and stiffness. To improve further, after 2 days he was initiated on baclofen 10 mg 3 times a day and gabapentin 300 mg twice a day. After 1 week, to help him further prevent attacks (for maintenance therapy), he was started on IVIG 0.4 g/kg per day for 5 days, equal to 2 g/kg. The patient's symptoms significantly improved, and he was able to ambulate after 1 week of treatment. Patient tachycardia also improved with GABAergic medications while the cardiologist decreased the metoprolol dose. The patient was referred to the university hospital for ongoing care following hospital discharge and to receive EMG as an outpatient.

## 3. Discussion

SPS is rare but also is underdiagnosed. Obtaining appropriate history and clinical exams is crucial, as well as ordering serum or CSF GAD, and confirmation by EMG. Differential diagnosis of SPS is broad and would include neuromuscular disorders, cervical myelopathy, multiple sclerosis, Parkinsonism, generalized anxiety disorder, and non-neurological diseases such as intra-abdominal pathologies and cardiac disease. On the other hand, there are patients with primary psychiatric disorders that get misdiagnosed with SPS [11]. Response to benzodiazepines can be seen in both SPS and anxiety disorder [12].

Severe weight loss in our patients seems to be a rare manifestation of SPS. One patient who was initially diagnosed with anorexia nervosa was found to have SPS [11]. The mechanism of weight loss can be fear of eating because of abdominal pain or GI dysfunction.

Mitsumoto described two SPS patients with sudden death due to paroxysmal autonomic dysfunction, highlighting the importance of early diagnosis of SPS patients with severe tachycardia [8]. Other features that can help for diagnosis include exaggerated startle response, unexplained falls, hyperreflexia, lumbar hyperlordosis, hypertonia, and moderate improvement with benzodiazepines [2, 3]. An abnormal MRI of paraspinal muscles with T2 hyperintensities/edema, although nonspecific, can be helpful to rule out functional cases.

Ordering GAD antibodies is important although at a low titer (< 2000 IU/mL) can be seen in nonspecific conditions such as diabetes. In our patient, the level was very high (> 25,000 IU/mL), not consistent with diabetes. EMG was not done but clinical exams including stiffness, thoracoabdominal spasms, high levels of GAD 65, and excluding other causes are likely consistent with SPS. Other features in our patient, including falls, lumbar hyperlordosis, and response to diazepam, are confirming the diagnosis.

SPS is a progressive neurological disease; without treatment, it could be disabling. As mentioned, presentation with cardiac symptoms can be more critical due to the possibility of sudden death. Initial treatment includes GABAergic-enhancing medications such as diazepam at 5–10 mg twice a day, and if tolerated, higher dose. Clonazepam 0.5–1 mg twice or three times a day is preferred by some neurologists. The baclofen starting dose is 10 mg three times a day and can be slowly increased to 20 mg three times a day if needed. Gabapentin is not a GABAergic medication but can be very helpful for painful spasms [2, 10]. Long-term maintenance therapy includes IVIG and other immunotherapies. In 67% of the patients, there was a meaningful response over a period of 3.3 years at 2 g/kg/month. If the patient does not respond to IVIG, consider rituximab and plasma exchange [2, 10].

## 4. Conclusion

SPS should be considered in the differential diagnosis of chronic abdominal, chest and back pain; tachycardia; and weight loss. High suspicion of clinical diagnosis includes painful spasms, stiffness, serological, and EMG findings, and, if needed, CSF analysis. Patients can present with predominantly non-neurological symptoms such as recurrent chest pain, abdominal pain, and tachycardia. Non-neurologists should be familiar with this disease to avoid severe disability or even death since this is a potentially treatable disease.

## Data Availability

The data used to support the findings of this study are available on request from the corresponding author.
